# Indefinite Statements

**Published:** 1871-11

**Authors:** 


### INDEFINITE STATEMENTS.

Ideas, to be valuable, must be conveyed in definite, precise, and full statements. A man takes half a column of a newspaper to say that the

GAPES IN CHICKENS, caused by one or more little white worms in the windpipe, can be always and promptly cured by killing the worms, which he does by feeding them on copperas, which is the sulphate of iron. He says, let the copperas stand in water, use that water to mix up the meal, and feed it to the chickens; it is certain death. But to the chicken or the worm? Copperas won't "stand in water," it melts rapidly. Must copperas be put in the water until the water will dissolve no more of it, thus making a saturated solution of copperas and water? if so, how much of the water to how much meal? Copperas is a corrosive poison; if a man were to take a swallow of this saturated solution, he would be pretty certain to die by its destroying the membrane or skin of the throat, and violent inflammation; about as certainly as by a drink of boiling water. Persons using it for chickens will have to feel their way along, but while they are doing that, the present brood of chickens will die off, for the gapes kill in a few days; but the experience gained may be used to advantage on the next generation of chicks. He says it will never fail to cure

HOG CHOLERA,

And horses too. Does he mean that it is a certain cure for hog cholera in horses? Perhaps hog cholera is allied in its nature to Asiatic cholera, and as both are supposed by some to be the result of multitudes of living things, perhaps copperas may be found to be a cure for all wormy people.

### TAPE WORMS AND PIN WORMS.

There is sometimes a very inconvenient itching at the extremity of the lower bowels, commencing soon after getting warm in bed at night. With some it is almost intolerable, lasting for hours. This is sometimes relieved instantly by using a short, small ivory syringe to throw a teaspoonful of camphor water just within the bowel called RECTUM by the doctors. Where this fails, the copperas might be tried, for this itching arises from the presence of a small worm. In case the camphor water, which all druggists keep constantly on hand, fails, put into about half a tablespoon of water a piece of copperas as large as half a Windsor bean, or larger than two peas. When dissolved, draw it up in the syringe and deposit it as before. Any person can make the camphor water by putting a piece of camphor in a bottle of water; that is, not to fall into the error complained of in the first part of the article, the camphor should be as large as a lump of sugar, and the bottle should be small! that is, a Windsor-bean sized piece of camphor into a bottle holding a tablespoonful or two of water.

